# Supplementary material for: Novel Driver Strength Index highlights important cancer genes in TCGA PanCanAtlas patients
Source: PeerJ. 2022 Aug 11;10:e13860. doi: 10.7717/peerj.13860 (PMC9375969; doi:10.7717/peerj.13860)
Supplement: Supplemental Information 2 [file peerj-10-13860-s002.zip › COHORTS/genes plots/cohorts/2021_8_16_14_9_distribution_cohorts_females_LGG.pdf]

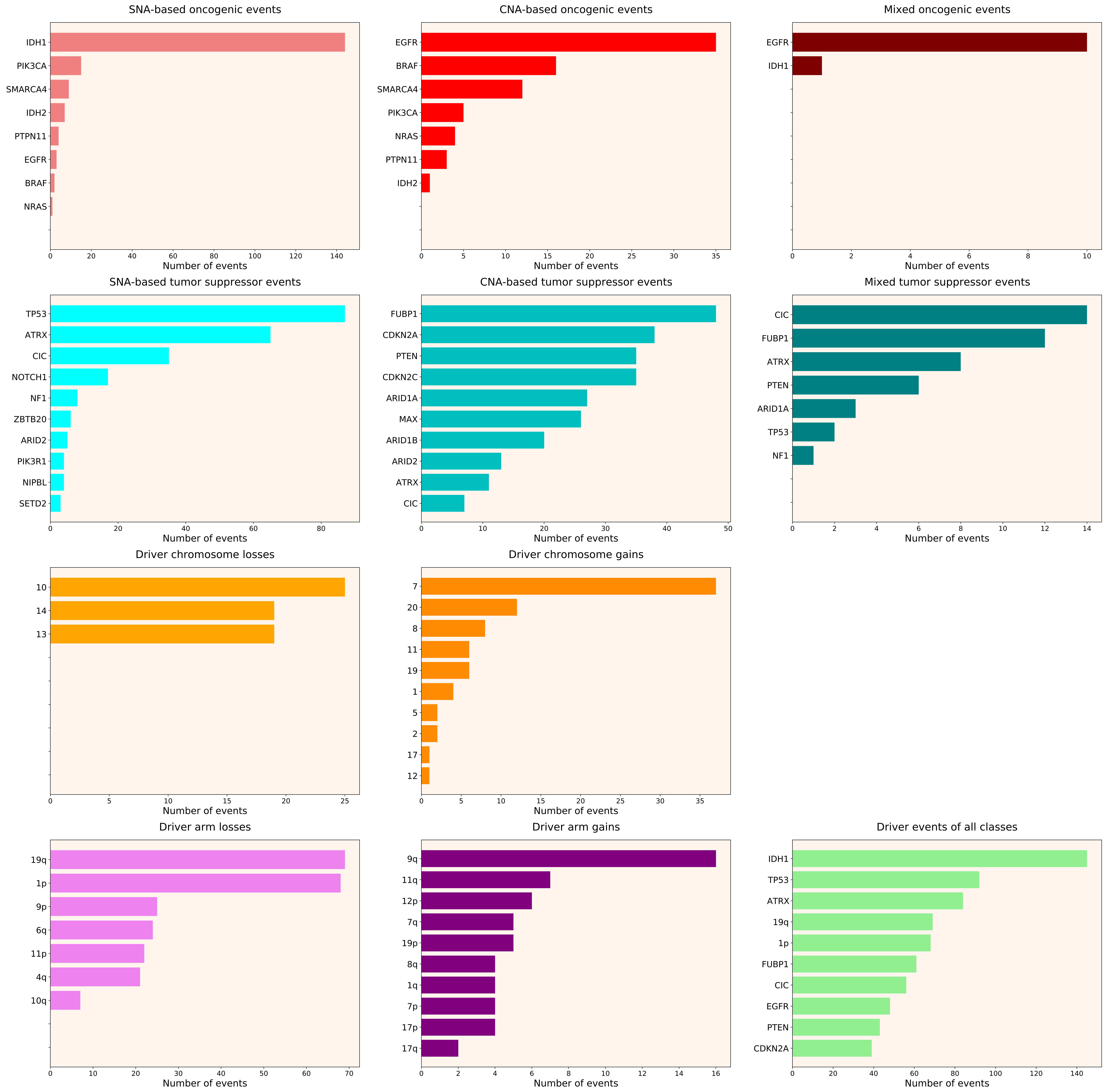
SNA-based oncogenic events
IDH1
PIK3CA
SMARCA4
IDH2
PTPN11
EGFR
BRAF
NRAS
0 20 40 60 80 100 120 140
Number of events
CNA-based oncogenic events
EGFR
BRAF
SMARCA4
PIK3CA
NRAS
PTPN11
IDH2
0 5 10 15 20 25 30 35
Number of events
Mixed oncogenic events
EGFR
IDH1
0 2 4 6 8 10
Number of events
SNA-based tumor suppressor events
TP53
ATRX
CIC
NOTCH1
NF1
ZBTB20
ARID2
PIK3R1
NIPBL
SETD2
0 20 40 60 80
Number of events
CNA-based tumor suppressor events
FUBP1
CDKN2A
PTEN
CDKN2C
ARID1A
MAX
ARID1B
ARID2
ATRX
CIC
0 10 20 30 40 50
Number of events
Mixed tumor suppressor events
CIC
FUBP1
ATRX
PTEN
ARID1A
TP53
NF1
0 2 4 6 8 10 12 14
Number of events
Driver chromosome losses
10
14
13
0 5 10 15 20 25
Number of events
Driver chromosome gains
7
20
8
11
19
1
5
2
17
12
0 5 10 15 20 25 30 35
Number of events
Driver arm losses
19q
1p
9p
6q
11p
4q
10q
0 10 20 30 40 50 60 70
Number of events
Driver arm gains
9q
11q
12p
7q
19p
8q
1q
7p
17p
17q
0 2 4 6 8 10 12 14 16
Number of events
Driver events of all classes
IDH1
TP53
ATRX
19q
1p
FUBP1
CIC
EGFR
PTEN
CDKN2A
0 20 40 60 80 100 120 140
Number of events
